# Gene Expression Profiling in the Cortex of *Fabp4* Knockout Mice

**DOI:** 10.1002/npr2.70006

**Published:** 2025-02-08

**Authors:** Hinako Kirikae, Xiaofeng He, Tetsuo Ohnishi, Hirofumi Miyazaki, Takeo Yoshikawa, Yuji Owada, Motoko Maekawa

**Affiliations:** ^1^ Department of Organ Anatomy Tohoku University Graduate School of Medicine Sendai Japan; ^2^ Department of Nutrition Akita Nutrition Junior College Akita Japan; ^3^ Laboratory for Molecular Psychiatry RIKEN Center for Brain Science Wako Saitama Japan

**Keywords:** autism spectrum disorder, epigenetic gene expression, *Fabp4* knockout (KO) mice, RNA‐seq, synaptic gene expression

## Abstract

**Aims:**

Fatty acid binding protein 4, adipocyte (*Fabp4*), is well known for its role in peripheral lipid metabolism, but its potential role in brain function remains largely unexplored. This study aimed to investigate *Fabp4* expression in the adult mouse brain and explore gene expression changes in *Fabp4* knockout (KO) mice to assess its potential impact on brain function.

**Methods:**

We conducted in situ hybridization to assess *Fabp4* expression in key brain regions of adult mice. In parallel, differential gene expression analysis using RNA‐seq was conducted in the prefrontal cortex of *Fabp4* KO mice to identify genes affected by *Fabp4* deficiency.

**Results:**

No *Fabp4* expression was detected in the brains of mice, suggesting a lack of direct involvement in the central nervous system. However, *Fabp4* KO mice exhibited significant changes in gene expression in the brain, with 31 genes upregulated and 30 downregulated. Downregulated genes were linked to histone methylation and metabolic processes, while upregulated ones were associated with synaptic organization.

**Conclusion:**

Although *Fabp4* is not expressed in the brain, its deficiency leads to substantial changes in gene expression, likely mediated by peripheral metabolic pathways and epigenetic regulation. These changes may explain the previously observed autism‐like behaviors and increased dendritic spine density in *Fabp4* KO mice. This study sheds light on the role of systemic lipid metabolism in neurodevelopmental disorders such as autism spectrum disorder (ASD) and highlights epigenetic mechanisms as potential mediators of these effects.

## Abstract 2

This study investigates the role of *Fabp4* in brain function using *Fabp4* knockout (KO) mice. Despite negligible *Fabp4* expression in the brain, RNA‐seq analysis revealed significant changes in genes associated with synaptic organization and epigenetic regulation. These findings support the concept of an “adipo‐brain axis,” where peripheral metabolic processes influence neural function and behavior.

## Graphical abstract

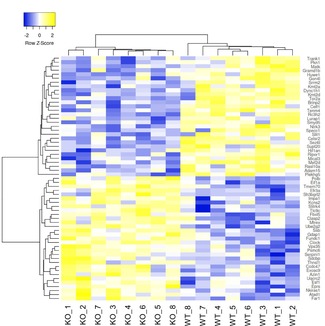

## 1 Introduction

Autism spectrum disorder (ASD) is a neurodevelopmental condition characterized by deficits in social communication, repetitive behaviors, and abnormal sensory processing. Epidemiological studies have identified links between ASD and metabolic conditions, particularly obesity and lipid metabolism disorders [1, 2]. Additionally, genome‐wide association studies and investigations of genetic mutations have further implicated lipid metabolism pathways in increasing the risk of ASD [3, 4, 5, 6]. These findings suggest that disruptions in lipid metabolism may play a role in ASD pathophysiology.

Fatty acid binding proteins (FABPs) are a family of low‐molecular‐weight proteins (~15 kDa) that play critical roles in fatty acid metabolism. Each of the 12 identified FABP isoforms is primarily expressed in specific tissues [7, 8]. Fatty acid binding protein 4, adipocyte (FABP4), or adipocyte‐type FABP (A‐FABP), is predominantly expressed in adipocytes and macrophages, where it regulates lipid storage, transport, and degradation. Elevated levels of circulating FABP4 are associated with various metabolic disorders, including atherosclerosis, insulin resistance, Type 2 diabetes, and cardiovascular diseases, as well as conditions such as asthma and cancer [9]. These associations indicate that FABP4 plays a significant role in lipid metabolism and related metabolic conditions. On the other hand, suppression or loss of FABP4 function has been reported to exert protective effects against dyslipidemia [10], suggesting that elevated and deficient FABP4 levels may exert opposing effects on lipid metabolism.

Several studies have reported a link between ASD and abnormal lipid metabolism [11, 12, 13]. Given the established associations between ASD and lipid metabolism abnormalities, as well as the critical role of FABP4 in lipid metabolism, we investigated the potential link between ASD and FABP4. Our previous research showed reduced plasma FABP4 levels in children with ASD and identified a family in which neuropsychiatric symptoms co‐segregate with an *FABP4* loss‐of‐function mutation. Furthermore, *Fabp4* knockout (KO) mice exhibited ASD‐like behaviors and increased dendritic spine density in pyramidal cells, mirroring findings from ASD postmortem brains [14]. Based on these findings, we proposed the concept of the adipo‐brain axis, in which metabolic processes in adipose tissue might influence neurodevelopment and behavior.

In this study, we examine gene expression changes in the brains of adult *Fabp4* KO mice to gain new insights into the consequences of FABP4 deficiency and its potential role in neural function and metabolism.

## 2 Materials and Methods

### 2.1 Animals and Housing Conditions

Inbred C57BL/6J mice were purchased from The Jackson Laboratory, Japan (Kanagawa, Japan). *Fabp4* KO mice, which have a C57BL/6J background, were generously provided by Prof. Hotamisligil [15]. *Fabp4* KO mice were maintained as a closed colony. Heterozygous *Fabp4* KO mice were intercrossed to generate wild‐type (WT) and *Fabp4*‐null mice. The animals were housed in groups of four or five in standard cages under temperature‐ and humidity‐controlled conditions, with a 12‐h light/dark cycle (lights on at 08:00), and had ad libitum access to their respective chow and tap water. All animal experiments were conducted using male mice. The experimental procedures were approved by the RIKEN Animal Ethics Committee (Approval Number: H30‐B030139) and the Center for Laboratory Animal Research, Tohoku University (Animal Experiment Approval Numbers 2021 ido‐067 and 2023 ido‐072).

### 2.2 In Situ Hybridization (ISH)

Eight‐week‐old C57BL/6J mice were deeply anesthetized with isoflurane and subsequently perfused transcardially with 4% paraformaldehyde (PFA) in 0.1 M PBS. The brains were postfixed in 4% PFA overnight, followed by dehydration and embedding in paraffin. Tissue sections were then sectioned into 4‐μm‐thick slices. Following the manufacturer's instructions, ISH was performed using the RNAscope 2.5 HD Reagent Kit‐RED (Advanced Cell Diagnostics Inc., Hayward, CA). The sections were hybridized with a target *Fabp4* probe (Accession Number: NM_024406.3; Target Region: 2–675; and ACD: 884621) at 40°C for 2 h. For quality control, a negative control probe targeting *DapB* (a bacterial gene, negative control; ACD: 310043) and a positive control probe targeting *Ppib* (peptidylprolyl isomerase B, a housekeeping gene; ACD: 313911) were also included. Both control probes were processed under identical conditions to validate the hybridization procedure. Slide imaging was conducted using the SLIDEVIEW VS200 slide scanner (EVIDENT, Tokyo, Japan).

### 2.3 RNA‐Seq Analysis

A comparative RNA‐seq analysis was conducted on 16‐week‐old *Fabp4* KO mice (*n* = 8) and their WT littermates (*n* = 8). We selected 16‐week‐old mice because our previous behavioral studies demonstrated that *Fabp4* KO mice display ASD‐like behavioral traits [14]. For further experimental details, see Appendix S1. Data matrices were processed and normalized for quality control using EdgeR with log2‐transformed counts per million (CPM+4) through the iDEP2.01 online analysis platform (http://bioinformatics.sdstate.edu/idep/), which is based on the R programming language. The statistical method DESeq2 was used to identify differentially expressed genes (DEGs) by comparing the two groups. Genes with a false discovery rate (FDR) < 0.1 and a fold change (FC) ≥ 1.1 were considered significant. Gene lists were further analyzed using bioinformatics tools to explore changes in biological pathways and networks, including the enrichment analysis function of iDEP2.01 and SynGO (Synaptic Gene Ontologies; https://www.syngoportal.org/), version 1.2. Heatmaps of the DEGs were generated using Heatmapper (http://www.heatmapper.ca/), with hierarchical clustering performed based on Pearson correlation.

## 3 Results

### 3.1 *Fabp4* Expression in the Mouse Brain

**FIGURE 1:**
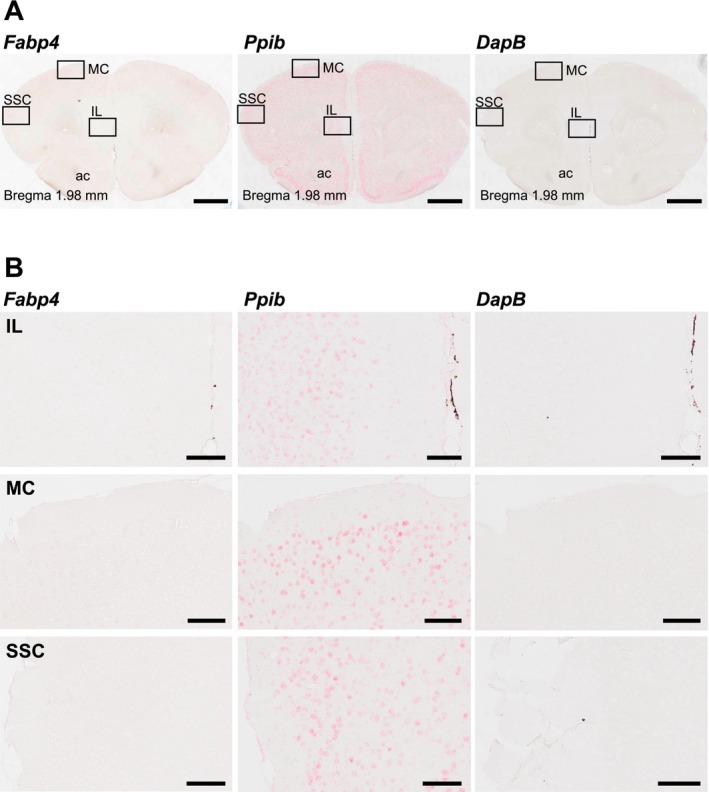
In situ hybridization analysis of *Fabp4* expression in the PFC. (A) Coronal sections at Bregma 1.98 mm show the prefrontal cortex (PFC), with the infralimbic cortex (IL), motor cortex (MC), and somatosensory cortex (SSC) regions outlined. These panels are shown: *Fabp4* probe (left), positive targeting *Ppib* (center), and negative control probe targeting *DapB* (right). No detectable *Fabp4* expression is observed in the PFC. The *Ppib* control shows a strong specific signal, confirming RNA integrity and successful hybridization, while the *DapB* control shows no signal, verifying the specificity of the experiment. (B) Higher‐magnification images of IL, MC, and SSC within the PFC. The layout follows the same order as in (A): *Fabp4* probe (left), positive control probe targeting *Ppib* (center), and negative control probe targeting *DapB* (right). No *Fabp4* signal was detected in any of these areas. Scale bars: 1 mm (A) and 100 μm (B).

To investigate the expression pattern of *Fabp4* in the brain, we conducted ISH on sections of the prefrontal cortex (PFC) from 8‐week‐old C57BL/6J mice, using probes targeting *Fabp4*, alongside negative (*DapB*) and positive (*Ppib*) controls. High‐magnification analysis of the infralimbic cortex (IL), somatosensory cortex (SSC), and motor cortex (MC) revealed no detectable *Fabp4* expression in these regions (Figure 1A,B). We extended the analysis to other brain regions, including the hippocampus and cerebellum, but similarly, no notable *Fabp4* mRNA signal was observed in these posterior brain regions (Figure S1A). These findings support previous research indicating that *Fabp4* is primarily expressed outside the brain [8, 16]. In contrast, robust *Fabp4* expression was observed in peripheral tissues such as adipose tissue and lungs (Figure S1B), consistent with its well‐established role in lipid metabolism and inflammation in these tissues.

### 3.2 Gene Expression in the Brain of *Fabp4* KO Mice

**FIGURE 2:**
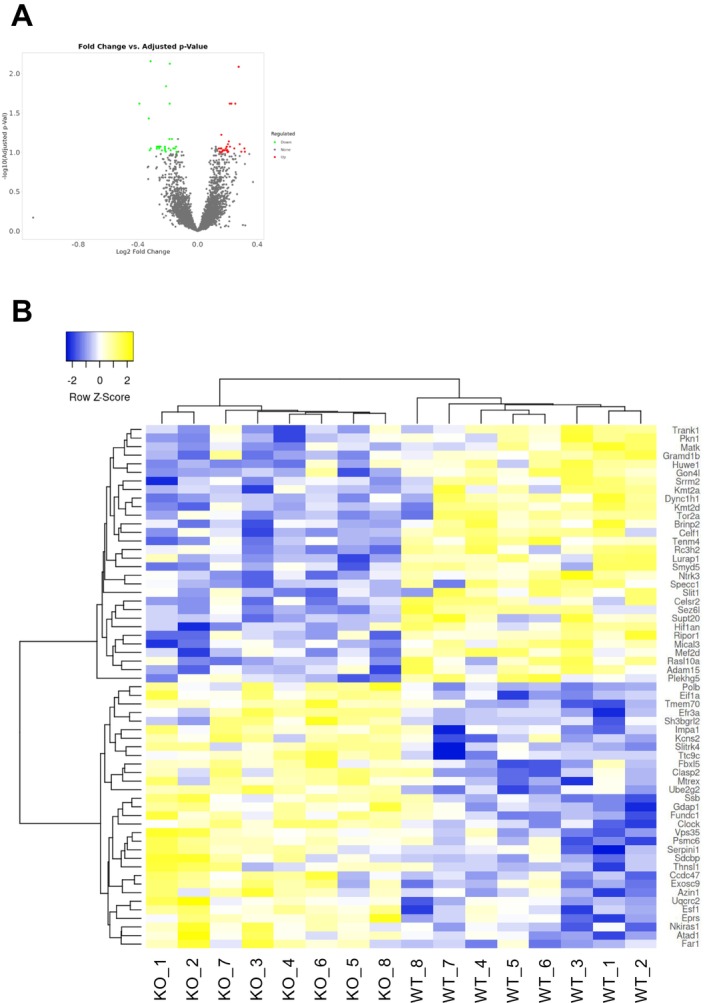
Volcano plots and heatmap representations of the differentially expressed gene (DEG) analysis. (A) Volcano plots depicting selected genes from two comparison groups. Green dots represent genes with decreased fold change (FC) in expression, while red dots indicate genes with increased FC. A total of 61 genes were identified with statistically significant expression changes. (B) Heatmap of significantly differentially expressed genes, with genes clustered based on expression levels. Blue boxes indicate genes with decreased expression, while yellow boxes represent genes with increased expression after log2 FC transformation. The clustering was performed using the Pearson correlation as the distance measurement method.

We conducted RNA‐seq analysis in adult PFC samples from *Fabp4* KO and WT mice (*n* = 8 each) to explore the molecular mechanisms underlying the observed ASD‐like phenotypes in *Fabp4*‐deficient mice [14]. Box plot and PCA (principal component analysis) of mRNA data confirmed that both groups were comparable (Figure S2A,B). DEG screening (FDR < 0.10; FC ≥ 1.1) identified 61 DEGs in the PFC of *Fabp4* KO mice compared to WT mice, with 31 genes upregulated and 30 genes downregulated (Figure 2A,B).

**FIGURE 3:**
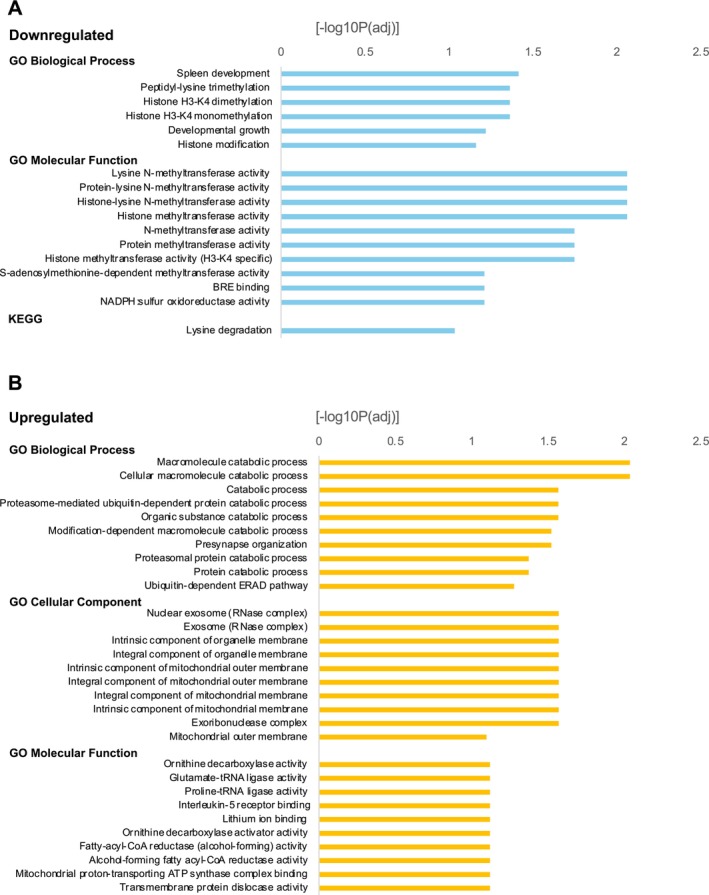
Gene Ontology (GO) pathway enrichment analysis of differentially expressed proteins (DEPs). (A) GO pathway enrichment analysis of downregulated DEPs. (B) GO pathway enrichment analysis of upregulated DEPs.

GO (Gene Ontology) and KEGG (Kyoto Encyclopedia of Genes and Genomes) enrichment analyses revealed that the downregulated DEGs were primarily associated with epigenetic modifications, including histone methylation. Notably, *Kmt2a* (*Mll1*), *Kmt2d* (*Mll2*), and *Huwe1*, all involved in histone methylation and ubiquitination, were among the downregulated genes. These genes are critical for regulating gene expression and chromatin structure. Additionally, downregulated DEGs were linked to metabolic and catalytic processes such as lysine degradation and oxidoreductase activity (Figure 3A).

The upregulated DEGs were enriched in catabolic processes related to protein degradation, including proteasome and ubiquitin‐dependent pathways. Furthermore, several DEGs were associated with synaptic organization and molecular functions, such as ornithine decarboxylase and fatty‐acyl‐CoA reductase activities (Figure 3B). These results suggest broad systemic changes in metabolism and synaptic organization in the PFC of the *Fabp4* KO mice, despite the lack of *Fabp4* expression in the brain.

### 3.3 Synapse‐Specific GO Analysis Using SynGO

**FIGURE 4:**
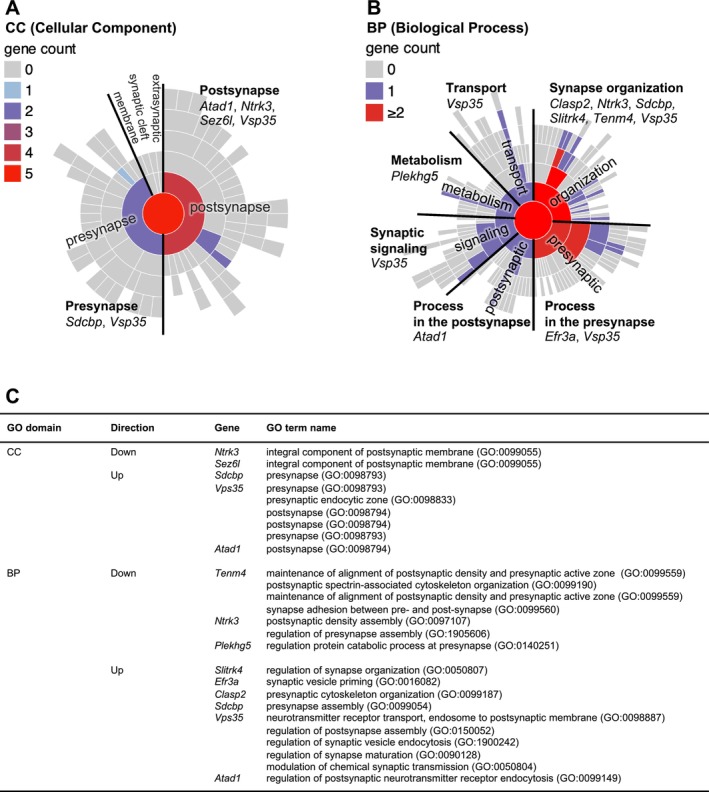
Synaptic gene annotation using SynGO. (A and B) Graphs from SynGO showing the number and names of differentially expressed genes (DEGs) included in GO terms related to synaptic function (A: cellular compartments; B: biological processes). A color‐coded legend indicates the number of genes in each GO term. (C) Table summarizing the GO domains, listing the GO terms, whether the corresponding genes were upregulated or downregulated, and the gene names.

Our previous study reported an increase in immature dendritic spines and a reduction in mature spines of pyramidal cells in the PFC of *Fabp4* KO mice, indicating possible disruptions in synaptic function [14]. To investigate whether these morphological changes in *Fabp4* KO mice were accompanied by alterations in synaptic gene expression, we performed a synapse‐specific GO analysis using the SynGO portal (https://syngoportal.org). SynGo provides high‐confidence functional annotations related specifically to synaptic components and processes, offering detailed insights into cellular component (CC) and biological process (BP) terms associated with synapses (Figure 4A,B).

The analysis revealed several downregulated genes involved in critical synaptic processes, including *Ntrk3* and *Sez6l*, both associated with postsynaptic signaling, *Tenm4*, essential for synaptic alignment, and *Plekhg5*, which regulates protein turnover at the presynaptic site (Figure 4C). These genes are key contributors to synaptic organization and stability, and their downregulation may explain the previously observed disruptions in spine maturation and synaptic integrity.

Among the upregulated genes, *Sdcbp* and *Clasp2* were linked to presynaptic assembly and cytoskeleton organization, and *Efr3a* was involved in synaptic vesicle priming. *Vps35* plays roles in both presynaptic and postsynaptic activities, including vesicle recycling, and *Atad1* was implicated in regulating postsynaptic receptor endocytosis, a process vital for synaptic plasticity. *Slitrk4*, another upregulated gene, contributes to synaptic organization and stability (Figure 4C). Collectively, these upregulated genes may represent compensatory responses that help maintain synaptic structure, regulate signaling, and ensure neurotransmitter release in the absence of *Fabp4*.

## 4 Discussion

In this study, we investigated the role of *Fabp4* in the adult mouse brain by examining its expression and the gene expression changes associated with its KO. Our findings show that while *Fabp4* is not observed in the brain, its absence leads to significant changes in gene expression, particularly in synaptic formation and epigenetic regulation.

The absence of *Fabp4* in WT brain tissue, confirmed through ISH, aligns with previous reports suggesting that *Fabp4* is primarily involved in peripheral lipid metabolism and is minimally expressed in the central nervous system [17, 18]. RNA‐seq analysis showed extremely low *Fabp4* transcript levels in the PFC of WT mice, with a mean TPM value of 0.13 across eight samples. In contrast, other FABP isoforms, such as *Fabp3*, *Fabp5*, and *Fabp7*, exhibited significantly higher expression levels (mean TPM values: 305.12, 255.16, and 107.43, respectively). These findings suggest that *Fabp4* expression in the brain is minimal, if present at all, and that the observed gene expression changes in *Fabp4* KO mice most likely result from systemic effects rather than direct involvement of *Fabp4* in neural tissue.

To further verify our observation, we reviewed public databases, including the Allen Brain Atlas (https://mouse.brain‐map.org/) and the Genotype‐Tissue Expression (GTEx) project (GTEx Analysis Release V10, dbGaP Accession phs000424.v10.p2, https://www.gtexportal.org/home/). Consistent with our findings, *Fabp4* expression values in various mouse brain regions, such as the isocortex, hippocampal formation, and cerebellum, were very low, ranging from 0.08 to 0.35 in the Allen Brain Atlas. Similarly, GTEx project indicated extremely low *FABP4* expression in human cortical regions, with nTPM values of 0.67 in Brodmann Area 24 and 0.77 in Brodmann Area 9. Among other brain regions, nTPM values slightly exceeding 1 were observed in the hippocampal formation and pituitary gland. However, these values remain relatively low.

Single‐nuclei analysis from another research group further confirmed negligible *Fabp4* expression in neuronal and glial cells, while detecting it in vascular‐associated cell types, such as choroid plexus epithelial cells, pericytes, and vascular smooth muscle cells [19]. Regarding fetal and developmental stages, data from Mousebrain.org (http://mousebrain.org/) indicate that *Fabp4* expression is minimal at embryonic day 10.5 (E10.5), with almost no detectable levels observed.

Taken together, these data support our conclusion that *Fabp4* is not expressed in the brain under normal conditions. However, we cannot completely rule out the possibility that transient *Fabp4* expression during specific developmental stages or in unknown minor cell populations plays a role in brain development. These findings align with our proposed concept of the “adipo‐brain axis.” According to this hypothesis, *Fabp4* primarily functions in peripheral tissues, such as adipose tissue, where it influences systemic metabolic processes that indirectly affect neural function and development. Our gene expression data potentially provide valuable clues to elucidate the molecular mechanisms by which *Fabp4* deficiency may affect brain function.

Through the general DEG analysis, several downregulated genes involved in epigenetic regulation were identified, such as *Kmt2a* (*Mll1*), *Kmt2d* (*Mll2*), and *Huwe1*, all of which are critical for histone methylation and gene regulation. The downregulation of *Kmt2a* is particularly of interest, given its role in maintaining prefrontal synaptic plasticity and working memory [20], with loss‐of‐function mutations in this gene being associated with ASD [20, 21]. *Kmt2d* is also essential for transcription during development, and its deficiency has been linked to developmental disorders [22]. The downregulation of *Huwe1*, a gene involved in neurodevelopment [23] and known to harbor *de novo* mutations in ASD [24], suggests potential disruptions in neuronal maintenance. Collectively, these changes may contribute to the ASD‐like phenotypes observed in *Fabp4* KO mice.

In addition to the general DEG analysis, we performed a synapse‐specific GO analysis using the SynGO portal to further investigate synaptic alterations in *Fabp4* KO mice. This targeted analysis identified multiple synapse‐related genes with altered expression in *Fabp4* KO mice. Among these, *Ntrk3* and *Sez6l*, critical components of the postsynaptic membrane [25, 26], are involved in synaptic signaling and receptor regulation, and *Tenm4* plays a crucial role in synaptic alignment [27, 28]. These genes are essential for maintaining the structural integrity of the synapse, and their downregulation may contribute to the previously observed changes in dendritic spine density in *Fabp4* KO mice [14].

Upregulated genes identified in the synapse‐specific GO analysis included *Sdcbp*, *Vps35*, *Atad1*, and *Slitrk4*, all of which are involved in both presynaptic and postsynaptic processes. *Vps35* plays a broad role in synaptic function, including vesicle recycling, receptor transport, and synapse maturation [29, 30], and *Atad1* regulates postsynaptic receptor endocytosis, a process essential for synaptic plasticity [31]. The upregulation of these genes may represent compensatory mechanisms in response to the synaptic deficits caused by *Fabp4* deficiency.

Although this study provides novel insights into the role of *Fabp4* in the brain, several limitations must be acknowledged. First, while we identified significant changes in gene expression, further research is needed to explore how these changes translate into functional alterations at the synaptic level. Additionally, the mechanisms through which *Fabp4* exerts indirect effects on brain function remain unclear. Future studies should investigate the adipo‐brain axis further, focusing on the systemic effects of *Fabp4* deficiency, including the potential involvement of peripheral lipid signaling or inflammatory pathways in modulating brain function. Moreover, while current data indicate that *Fabp4* is not expressed in the developing or adult brain under normal conditions, we cannot completely rule out the possibility of transient expression during specific developmental stages or in other ASD‐related brain regions not examined in this study. Future studies should explore these possibilities to fully understand the temporal and spatial dynamics of *Fabp4* expression and its potential impact on neurodevelopment.

In conclusion, while *Fabp4* is absent in the WT brain, its deficiency in *Fabp4* KO mice results in significant changes in gene expression, particularly in synaptic function and epigenetic regulation. These findings suggest that Fabp4/FABP4 plays an indirect yet crucial role in maintaining synaptic integrity, probably through systemic hypothesis we propose. This study provides valuable insights into the link between lipid metabolism and brain function, with potential implications for neurodevelopmental disorders such as ASD.

## Author Contributions

M.M. designed the study. H.X., H.K., H.M., and M.M. performed the experiments. H.X., H.K., and M.M. analyzed the data. T.O. and M.M. wrote the first draft of the manuscript. Y.O. and T.Y. edited the manuscript. All authors read and contributed to the final revision of the manuscript to ensure this is the case.

## Ethics Statement

All animal care and handling procedures were conducted in accordance with the “Guidelines for Animal Care and Use in RIKEN and Tohoku University.” All experimental protocols were approved by the RIKEN Animal Ethics Committee (Approval Number: H30‐B030139) and the Center for Laboratory Animal Research, Tohoku University (Animal Experiment Approval Numbers: 2021 ido‐067 and 2023 ido‐072).

## Consent

The authors have nothing to report.

## Conflicts of Interest

The authors declare no conflicts of interest.

## Supporting information


Figure S1.

Figure S2.

Appendix S1.

## Data Availability

The raw data obtained in this study are available on the DDBJ database (http://www.ddbj.nig.ac.jp/index‐e.html) under the BioProject accession number PRJDB19988 in the DDBJ BioProject database. BioSample metadata are available in the DDBJ BioSample database under accession numbers SAMD00875673–SAMD00875688.

## References

[1] A. P. Hill , K. E. Zuckerman , and E. Fombonne , “Obesity and Autism,” Pediatrics 136, no. 6 (2015): 1051–1061. 26527551 10.1542/peds.2015-1437 PMC4657601

[2] J. M. Gaspar , H. M. Carvalho , and A. Camacho‐Morales , “Editorial: Metabolic Disorders Associated With Autism Spectrum Disorders: Approaches for Intervention,” Frontiers in Neuroscience 15 (2021): 809978. 34924949 10.3389/fnins.2021.809978 PMC8675244

[3] M. Maekawa , Y. Iwayama , R. Arai , et al., “Polymorphism Screening of Brain‐Expressed FABP7, 5 and 3 Genes and Association Studies in Autism and Schizophrenia in Japanese Subjects,” Journal of Human Genetics 55, no. 2 (2010): 127–130. 20057506 10.1038/jhg.2009.133

[4] M. Maekawa , Y. Iwayama , T. Ohnishi , et al., “Investigation of the Fatty Acid Transporter‐Encoding Genes SLC27A3 and SLC27A4 in Autism,” Scientific Reports 5 (2015): 16239. 26548558 10.1038/srep16239 PMC4637822

[5] C. Shimamoto , T. Ohnishi , M. Maekawa , et al., “Functional Characterization of FABP3, 5 and 7 Gene Variants Identified in Schizophrenia and Autism Spectrum Disorder and Mouse Behavioral Studies,” Human Molecular Genetics 23, no. 24 (2014): 6495–6511. 25027319 10.1093/hmg/ddu369 PMC4240203

[6] I. Kushima , B. Aleksic , M. Nakatochi , et al., “Comparative Analyses of Copy‐Number Variation in Autism Spectrum Disorder and Schizophrenia Reveal Etiological Overlap and Biological Insights,” Cell Reports 24, no. 11 (2018): 2838–2856. 30208311 10.1016/j.celrep.2018.08.022

[7] A. W. Zimmerman and J. H. Veerkamp , “New Insights Into the Structure and Function of Fatty Acid‐Binding Proteins,” Cellular and Molecular Life Sciences 59, no. 7 (2002): 1096–1116. 12222958 10.1007/s00018-002-8490-y PMC11337517

[8] M. Furuhashi and G. S. Hotamisligil , “Fatty Acid‐Binding Proteins: Role in Metabolic Diseases and Potential as Drug Targets,” Nature Reviews. Drug Discovery 7, no. 6 (2008): 489–503. 18511927 10.1038/nrd2589 PMC2821027

[9] K. J. Prentice , J. Saksi , and G. S. Hotamisligil , “Adipokine FABP4 Integrates Energy Stores and Counterregulatory Metabolic Responses,” Journal of Lipid Research 60, no. 4 (2019): 734–740. 30705117 10.1194/jlr.S091793 PMC6446704

[10] K. Maeda , H. Cao , K. Kono , et al., “Adipocyte/Macrophage Fatty Acid Binding Proteins Control Integrated Metabolic Responses in Obesity and Diabetes,” Cell Metabolism 1, no. 2 (2005): 107–119. 16054052 10.1016/j.cmet.2004.12.008

[11] N. Usui , K. Iwata , T. Miyachi , et al., “VLDL‐Specific Increases of Fatty Acids in Autism Spectrum Disorder Correlate With Social Interaction,” eBioMedicine 58 (2020): 102917. 32739868 10.1016/j.ebiom.2020.102917 PMC7393524

[12] I. Bukelis , F. D. Porter , A. W. Zimmerman , and E. Tierney , “Smith‐Lemli‐Opitz Syndrome and Autism Spectrum Disorder,” American Journal of Psychiatry 164, no. 11 (2007): 1655–1661. 17974928 10.1176/appi.ajp.2007.07020315

[13] E. Tierney , N. A. Nwokoro , F. D. Porter , L. S. Freund , J. K. Ghuman , and R. I. Kelley , “Behavior Phenotype in the RSH/Smith‐Lemli‐Opitz Syndrome,” American Journal of Medical Genetics 98, no. 2 (2001): 191–200. 11223857 10.1002/1096-8628(20010115)98:2<191::aid-ajmg1030>3.0.co;2-m

[14] M. Maekawa , T. Ohnishi , M. Toyoshima , et al., “A Potential Role of Fatty Acid Binding Protein 4 in the Pathophysiology of Autism Spectrum Disorder,” Brain Communications 2, no. 2 (2020): fcaa145. 33225276 10.1093/braincomms/fcaa145 PMC7667725

[15] G. S. Hotamisligil , R. S. Johnson , R. J. Distel , R. Ellis , V. E. Papaioannou , and B. M. Spiegelman , “Uncoupling of Obesity From Insulin Resistance Through a Targeted Mutation in aP2, the Adipocyte Fatty Acid Binding Protein,” Science 274, no. 5291 (1996): 1377–1379. 8910278 10.1126/science.274.5291.1377

[16] G. S. Hotamisligil and D. A. Bernlohr , “Metabolic Functions of FABPs—Mechanisms and Therapeutic Implications,” Nature Reviews. Endocrinology 11, no. 10 (2015): 592–605. 10.1038/nrendo.2015.122 PMC4578711 26260145

[17] C. S. Heffner , C. Herbert Pratt , R. P. Babiuk , et al., “Supporting Conditional Mouse Mutagenesis With a Comprehensive Cre Characterization Resource,” Nature Communications 3, no. 1 (2012): 1–9. 10.1038/ncomms2186 PMC3514490 23169059

[18] X. Xiang , F. Yuan , J. Zhao , et al., “Deficiency in Pulmonary Surfactant Proteins in Mice With Fatty Acid Binding Protein 4‐Cre‐Mediated Knockout of the Tuberous Sclerosis Complex 1 Gene,” Experimental Physiology 98, no. 3 (2013): 830–841. 23143994 10.1113/expphysiol.2012.069674 PMC3593000

[19] K. Siletti , R. Hodge , A. Mossi Albiach , et al., “Transcriptomic Diversity of Cell Types Across the Adult Human Brain,” Science 382, no. 6667 (2023): eadd7046. 37824663 10.1126/science.add7046

[20] M. Jakovcevski , H. Ruan , E. Y. Shen , et al., “Neuronal Kmt2a/Mll1 Histone Methyltransferase Is Essential for Prefrontal Synaptic Plasticity and Working Memory,” Journal of Neuroscience 35, no. 13 (2015): 5097–5108. 25834037 10.1523/JNEUROSCI.3004-14.2015 PMC4380991

[21] S. De Rubeis , X. He , A. P. Goldberg , et al., “Synaptic, Transcriptional and Chromatin Genes Disrupted in Autism,” Nature 515, no. 7526 (2014): 209–215. 25363760 10.1038/nature13772 PMC4402723

[22] Z. Shan , Y. Zhao , X. Chen , et al., “KMT2D Deficiency Leads to Cellular Developmental Disorders and Enhancer Dysregulation in Neural‐Crest‐Containing Brain Organoids,” Science Bulletin (Beijing) 69, no. 22 (2024): 3522–3546. 10.1016/j.scib.2024.09.004 39327125

[23] A. C. Giles and B. Grill , “Roles of the HUWE1 Ubiquitin Ligase in Nervous System Development, Function and Disease,” Neural Development 15, no. 1 (2020): 6. 32336296 10.1186/s13064-020-00143-9 PMC7184716

[24] C. Nava , F. Lamari , D. Héron , et al., “Analysis of the Chromosome X Exome in Patients With Autism Spectrum Disorders Identified Novel Candidate Genes, Including TMLHE,” Translational Psychiatry 2, no. 10 (2012): e179. 23092983 10.1038/tp.2012.102 PMC3565810

[25] K. A. Han , D. Woo , S. Kim , et al., “Neurotrophin‐3 Regulates Synapse Development by Modulating TrkC‐PTPσ Synaptic Adhesion and Intracellular Signaling Pathways,” Journal of Neuroscience 36, no. 17 (2016): 4816–4831. 27122038 10.1523/JNEUROSCI.4024-15.2016 PMC6601719

[26] W. Q. Qiu , S. Luo , S. A. Ma , et al., “The Sez6 Family Inhibits Complement by Facilitating Factor I Cleavage of C3b and Accelerating the Decay of C3 Convertases,” Frontiers in Immunology 12 (2021): 607641. 33936031 10.3389/fimmu.2021.607641 PMC8081827

[27] T. J. Mosca , “On the Teneurin Track: A New Synaptic Organization Molecule Emerges,” Frontiers in Cellular Neuroscience 9 (2015): 204. 26074772 10.3389/fncel.2015.00204 PMC4444827

[28] W. Hong , T. J. Mosca , and L. Luo , “Teneurins Instruct Synaptic Partner Matching in an Olfactory Map,” Nature 484, no. 7393 (2012): 201. 22425994 10.1038/nature10926 PMC3345284

[29] Y. Tian , F. L. Tang , X. D. Sun , et al., “VPS35‐Deficiency Results in an Impaired AMPA Receptor Trafficking and Decreased Dendritic Spine Maturation,” Molecular Brain 8, no. 1 (2015): 70. 26521016 10.1186/s13041-015-0156-4 PMC4628247

[30] C. A. Kadgien , A. Kamesh , and A. J. Milnerwood , “Endosomal Traffic and Glutamate Synapse Activity Are Increased in VPS35 D620N Mutant Knock‐In Mouse Neurons, and Resistant to LRRK2 Kinase Inhibition,” Molecular Brain 14, no. 1 (2021): 1–20. 34530877 10.1186/s13041-021-00848-w PMC8447518

[31] L. Wang and P. Walter , “Msp1/ATAD1 in Protein Quality Control and Regulation of Synaptic Activities,” Annual Review of Cell and Developmental Biology 36 (2020): 141–164. 10.1146/annurev-cellbio-031220-015840 32886535

