# Supplementary figures and images for: Gene Expression Profiling in the Cortex of Fabp4 Knockout Mice
Source: Neuropsychopharmacol Rep. 2025 Feb 8;45(1):e70006. doi: 10.1002/npr2.70006 (PMC11806211; doi:10.1002/npr2.70006)

**A**

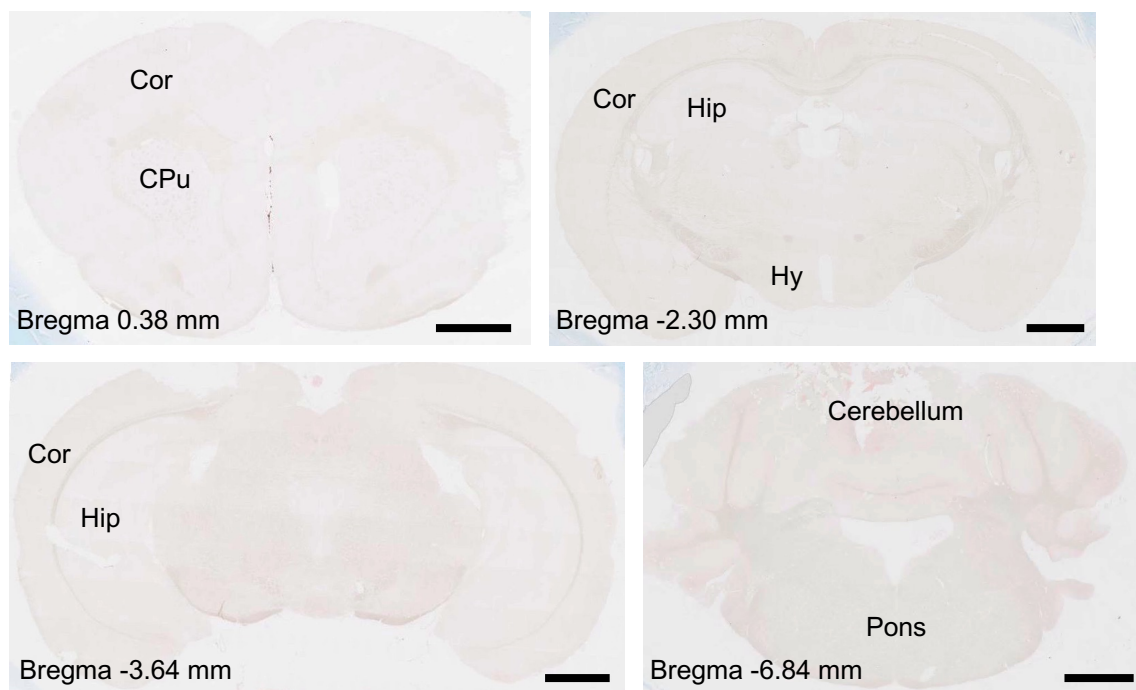

**B**

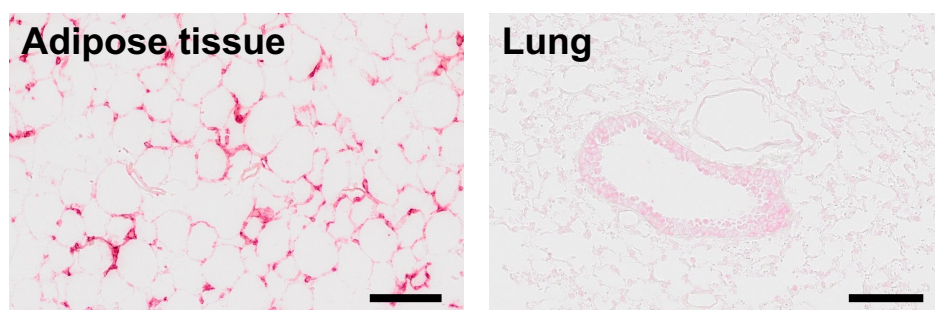

**Supplementary Figure 1**

Supplement: Supplementary file 1 — Figure S1. [file NPR2-45-e70006-s001.pdf]

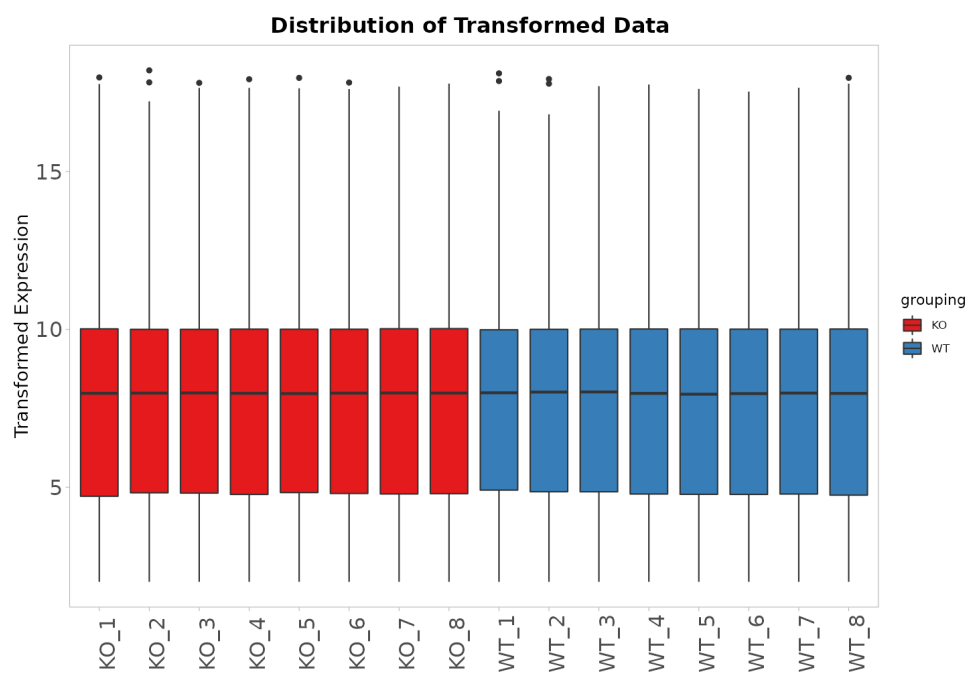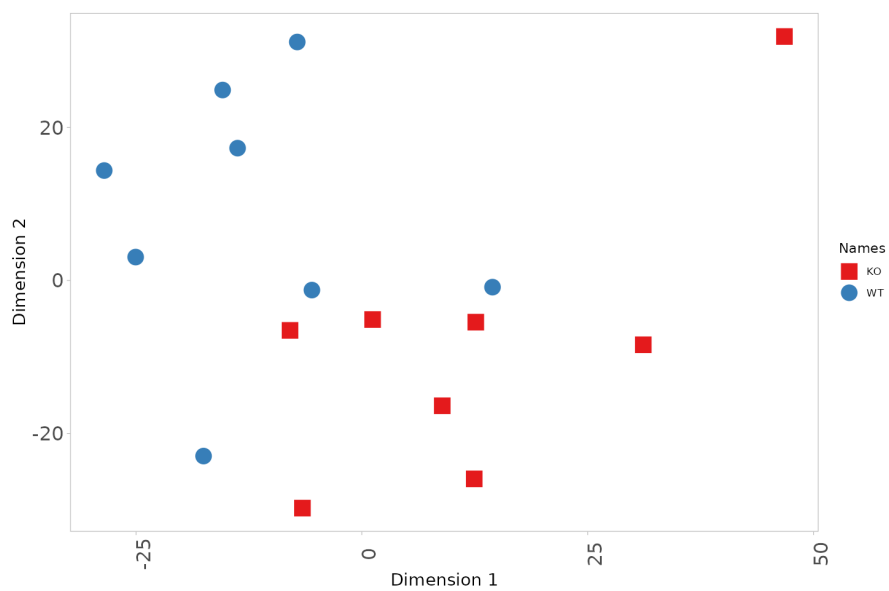

**Supplementary Figure 2**

Supplement: Supplementary file 2 — Figure S2. [file NPR2-45-e70006-s003.pdf]
