# Supplementary material for: Gene Expression Profiling in the Cortex of Fabp4 Knockout Mice
Source: Neuropsychopharmacol Rep. 2025 Feb 8;45(1):e70006. doi: 10.1002/npr2.70006 (PMC11806211; doi:10.1002/npr2.70006)
Supplement: Supplementary file 3 — Appendix S1. [file NPR2-45-e70006-s002.docx]

**SUPPLEMENTARY INFORMATION**

**Supplementary Figure 1:** *In situ* hybridization analysis of *Fabp4* expression. (**A**) Representative *in situ* hybridization images at Bregma 0.38, -2.30, -3.64, -6.84 mm, showing no detectable *Fabp4* mRNA in the cerebral cortex, hippocampus, amygdala, or cerebellum. (**B**) In contrast, clear *Fabp4* signals were observed in adipose tissue and the lungs. Scale bars: 1 mm (**A**); 100 μm (**B**).

**Supplementary Figure 2**: Bioinformatics analysis of RNA-seq data. (**A**) Box plot representing gene expression distribution in *Fabp4* knockout (KO) and wild-type (WT) mice. (**B**) Principal component analysis (PCA) shows the mRNA expression profile clustering between *Fabp4* KO and WT mice.

**Materials and Methods**

**RNA-seq analysis**

Total RNA was extracted from the prefrontal cortex using the miRNeasy Mini Kit (QIAGEN). cDNA libraries were generated from total RNA samples (2.0 μg, RNA integrity number (RIN) ≥ 8.7) using the TruSeq Stranded mRNA Sample Prep Kit, following the manufacturer's instructions (Illumina, San Diego, CA, USA). The quality of the cDNA libraries was assessed using a TapeStation (Agilent, Santa Clara, CA) or Bioanalyzer (Agilent). The cDNA libraries were sequenced using a 150-bp paired-end read format on the NovaSeq 6000 platform (Illumina). We utilized the DRAGEN Bio-IT Platform (Illumina) for this analysis, following the workflow outlined below. The sequence reads obtained from the sequencing analysis was mapped to the reference genome GRCm38.primary_assembly.genome.fa.gz and expression levels were calculated at both the gene and transcript levels using the gene annotation file gencode.vM25.primary_assembly.annotation.gtf.gz. The reference genome and gene annotation files were obtained from the GENCODE website (release m25). Annotation data were added, and the expression levels were summarized in a tabular format.
